# Fractionated stereotactic radiotherapy (fSRT) for symptomatic WHO grade 1 cavernous sinus meningiomas: long-term local control, clinical response, and toxicity outcomes

**DOI:** 10.1007/s11060-026-05688-z

**Published:** 2026-06-30

**Authors:** Ryan Shah, Shray Jain, Nilanjan Haldar, Lauren A. Holt, Robert L. Walker, Christopher J. Farrell, Debanjan Haldar, James J. Evans, Gregory S. Alexander, Wenyin Shi

**Affiliations:** 1 Sidney Kimmel Medical College, 1025 Walnut St, Philadelphia, PA 19107 USA; 2 Drexel University College of Medicine, 60 N 36th St, Philadelphia, PA 19104 USA; 3 Department of Radiation Oncology, Thomas Jefferson University Hospital, 111 S 11th St, Philadelphia, PA 19107 USA; 4 Department of Neurological Surgery, Thomas Jefferson University Hospital, 909 Walnut St, Philadelphia, PA 19107 USA

**Keywords:** Meningioma, Radiation, Cavernous sinus, Stereotactic, Benign

## Abstract

**Purpose:**

Cavernous sinus meningiomas represent a significant clinical challenge, given the high prevalence of neurological deficits and limited surgical options. Radiation therapy is often the preferred treatment. This study evaluates the effectiveness of fractionated stereotactic radiation therapy (fSRT) in achieving local control and symptomatic relief in patients with cavernous sinus meningiomas.

**Methods:**

This is a single-institution retrospective analysis of patients with symptomatic presumed WHO grade 1 cavernous sinus meningiomas treated with fSRT (1.8–2 Gy per fraction). Clinical data were collected by chart review. Kaplan-Meier curves were generated to assess local control. Clinical responses were evaluated for symptom improvement, stability, or worsening at ≤ 36 months, 36–60 months, 60–120 months, 120–180 months, and > 180 months.

**Results:**

This study identified 39 patients treated with fSRT who met inclusion criteria. Median age was 60 years (range 21–79), with a median Karnofsky Performance Score (KPS) of 90. Median follow-up time was 122.2 months. Median tumor volume was 10.0 cc (range 3.6–18.3 cc). Local control rates at 1, 3, 5, and 10 years were 100%, 94.6%, 91.7%, and 88.1%, respectively. Of 39 patients, 26 (66.7%) demonstrated clinical improvement following fSRT, 9 (23.1%) remained clinically unchanged, and 4 (10.3%) experienced worsening.

**Conclusion:**

Our study demonstrated fSRT achieves effective local control for cavernous sinus meningioma. Notably, 2/3 of patients achieved neurological symptom improvement after radiation. Larger, prospective studies are warranted to validate these findings.

## Introduction

Meningiomas are the most common primary intracranial neoplasm diagnosed in adults, making up 40% of primary brain tumor diagnoses [1]. Incidence of meningiomas increases with age, with over half occurring in patients over 65 years old [1–3]. Additionally, a 2:1 ratio of female predominance, along with high levels of progesterone, estrogen, and androgen receptor expression, suggests that hormones play a crucial role in meningioma pathophysiology [4]. Over 80% are benign, low-grade tumors, characterized by slow growth at a rate of about 1 mm³ per year [5–8]. Consequently, many meningiomas may go undetected for years, not causing symptoms until reaching significant size and/or mass effect [7, 8]. However, the timing and severity of reported symptoms varies significantly by tumor location, and even low-grade meningiomas can cause significant morbidity and mortality through compression of critical brain structures [9]. In particular, up to 2% of intracranial meningiomas involve the cavernous sinus, which can cause symptoms related to compression of neurovascular structures within the sinus, including cranial nerves III through VI and the internal carotid artery [10].

For symptomatic WHO Grade 1 meningiomas, maximal safe surgical resection is the preferred primary local treatment for symptomatic meningiomas [11]. Achieving gross total resection (GTR) can be challenging in cavernous sinus meningiomas, as surgeons must be cautious around neurovascular structures. In the literature, reported rates of GTR vary widely from 12 to 80%, and rates of cranial neuropathy recovery after surgery range from 14–67% [12].

Radiation therapy (RT) may serve as either an adjunct to subtotal resection (STR) or as definitive therapy for cavernous sinus meningioma. Stereotactic radiosurgery (SRS) with a single dose from 12 to 16 Gy has demonstrated excellent local control rates exceeding 85–90% [13]. However, SRS may not be feasible for larger tumors due to increased risk of toxicity when lesions abutting sensitive structures [14]. The risk of permanent complications, most commonly cranial neuropathies, following SRS for cavernous sinus meningiomas ≥ 10 cm³ may be up to 21% [14]. For larger tumors and those near neurovascular structures, fractionated stereotactic radiotherapy (fSRT), typically 1.8–2 Gy over 25–30 fractions to a total dose of 50–54 Gy, offers an effective approach with reduced risks compared to SRS [15]. Long-term local control rates with fSRT for cavernous sinus meningiomas consistently exceed 85% at 5 and 10 years, with a superior complication profile compared to SRS [15].

While the current literature contains favorable evidence for the efficacy of fSRT in treating WHO Grade 1 cavernous sinus meningiomas, few studies contain long-term follow-up data for both radiographic and clinical outcomes. The purpose of this single-institution retrospective cohort study is to report long-term outcomes, including local control, symptom response, and treatment-related toxicity, for a cohort of patients with symptomatic cavernous sinus meningiomas treated with fSRT.

## Methods

### Study design

This single-center retrospective cohort study of patients received fSRT for meningiomas involving the cavernous sinus between January 2006 and December 2017 at Sidney Kimmel Comprehensive Cancer Center, Thomas Jefferson University. This study was approved by the Institutional Review Board at Sidney Kimmel Cancer Center, with a waiver of informed consent.

### Inclusion criteria

Inclusion criteria were: (1) adult patients with cavernous sinus meningioma, with pathological diagnosis or imaging consistent with WHO grade 1 intracranial meningioma; (2) presence of symptoms attributable to the tumor; (3) treatment with fSRT delivering 1.8–2.0 Gy per fraction (50–54 Gy over 25–30 fractions); (4) availability of treatment and follow-up records, with a minimum of 1-year follow-up.

Imaging consistent with WHO grade 1 meningioma included a dural-based mass with homogeneous enhancement on post-contrast T1-weighted MRI, and the absence of malignant features. Inclusion without histological confirmation required these features, along with consensus by a multidisciplinary tumor board.

Exclusion criteria included meningiomas not involving the cavernous sinus, atypical and non-WHO Grade 1 meningiomas, and prior cranial radiation treatment.

### Radiation technique

**Fig. 1:**
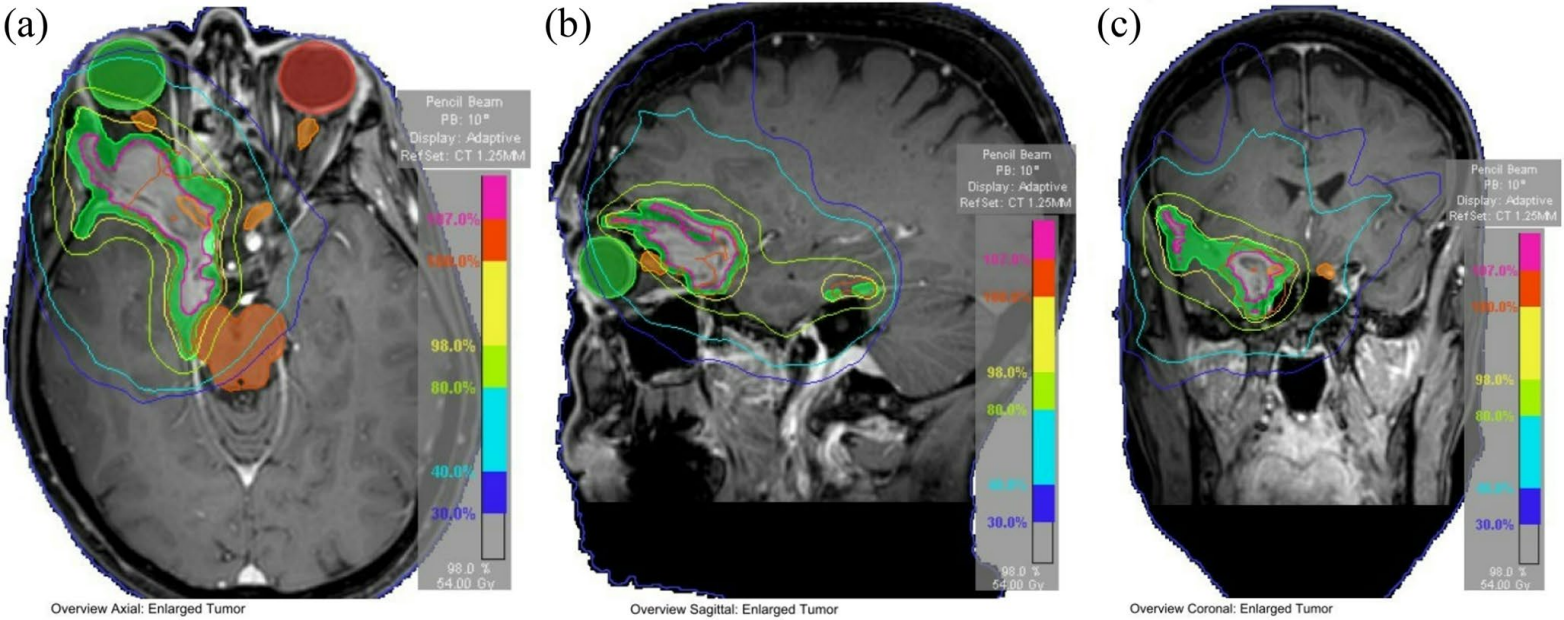
Sample fSRT plan for patient presenting with G1 cavernous sinus meningioma treated with 54 Gy in 30 fractions, with (**a**) axial, (**b**) sagittal, and (**c**) coronal views

All patients had thin cut (1–1.5 mm) axial post-contrast MRI. All underwent computed tomography (CT) simulation with Brainlab stereotactic head mask (Brainlab. Munich, Germany). MRI and CT images were fused for target and organ at risk (OAR) contouring. The gross tumor volume (GTV) was defined as the gross enhancing tumor on postcontrast isotropic 3-dimensional MRIs. The PTV (planning target volume) was created by adding a 1–2 mm isotropic margin to the GTV. Key OARs include brainstem, eyes, lens, optic nerves, and optic chiasm. Patients were treated to a prescription dose ranging from 50.4 to 54 Gy, delivered in 25 to 30 daily fractions (1.8–2.0 Gy per fraction). The most common prescription dose was 54 Gy over 30 fractions. In some cases, dose fractionation was individualized based on patient logistical challenges necessitating fewer fractions or tumor proximity to the optic apparatus, consistent with National Comprehensive Cancer Network (NCCN) guidelines recommending that the standard 54 Gy for WHO grade 1 meningiomas may be reduced to 50–50.4 Gy near critical organs at risk [16]. Patients were treated using intensity-modulated radiation therapy (IMRT) technique or volumetric Modulated Arc Therapy (VMAT) (Fig. 1). Treatment planning was carried out with Brain Lab iPlan (Brainlab, Munich, Germany), or Eclipse (Varian, Palo Alto, CA). Radiation treatment was delivered with Truebeam STx (Varian, Palo Alto, CA) with HD MLC (high definition multileaf collimator) and daily exacTRAC (Brainlab, Munich, Germany) on board imaging.

### Outcome measures

Patient demographic information, including age, sex, race/ethnicity, Karnofsky performance status (KPS), symptoms at presentation, and history of prior surgery, were collected from medical records. Dosimetric characteristics, including total dose, fractionation, and target volume, were recorded.

Post-fSRT follow-up, which continued for at least 1 year, involved clinical evaluation and contrast-enhanced MRI. Clinical response was categorized as “improved,” “unchanged,” or “worsened” based on clinical documentation from follow-up visits. Symptom response was abstracted from clinical documentation by a single investigator. Objective exam documentation was prioritized for classifying symptom response, but subjective descriptions were also considered. Improvement was not required to persist on subsequent visits, but documented regression of symptom improvement was categorized as “worsened” for that subsequent time bin. Patients with multiple presenting symptoms were classified based on improvement or worsening of any symptom, with no patients demonstrating simultaneous improvement and worsening of different symptoms. Radiographic response was assessed by Response Evaluation Criteria in Solid Tumors (RECIST v1.1), applied using the longest axial diameter of the enhancing tumor on post-contrast T1-weighted MRI, in accordance with institutional practice during the study period. Local control (LC) was defined as radiographic stability or decrease in tumor size. Measurements were abstracted from radiology reports and source imaging by a single investigator and reviewed by the treating radiation oncologist; formal inter-reader reliability was not assessed. Follow-up data were sorted into the following time bins: ≤36 months, 36–60 months, 60–120 months, 120–180 months, and > 180 months.

Treatment-related toxicity was graded using the Common Terminology Criteria for Adverse Events (CTCAE), version 5.0. Radiation necrosis was confirmed by MRI and clinical documentation, then graded by CTCAE criteria.

### Statistical analysis

Statistical analyses were planned a priori. Descriptive statistics were used to summarize patient, tumor, and dosimetric characteristics and outcomes. Actuarial rates of LC were calculated using the Kaplan-Meier method, with time zero being the date of the first treatment of fSRT. Statistical significance was set at *p* < 0.05.

## Results

### Patient characteristics

A total of 57 patients received fSRT for cavernous sinus meningiomas, of which 39 were included. Ten patients were excluded for insufficient follow-up of at least 12 months, 6 due to prior RT, and 2 due to no presenting symptoms at the time of diagnosis. Among the final cohort of 39 patients, the median age was 60 years (range 21–79 years), and 30 were female (71.4%) (Table 1). The median pre-treatment Karnofsky Performance Status was 90 (range 50–100). Fourteen patients (35.9%) had prior surgery. The most common neurological symptoms at presentation were diplopia (53.8%), CN (cranial nerve) VI palsy (41.0%), CN III palsy (30.8%), and headache (28.2%) (Table 2).

**Table 1:** Patient characteristics (*n* = 39)

Demographics	
Age (median years, range)	60 (21–79)
Sex	
Female (%)	27 (69.2)
Male (%)	12 (30.8)
**Race (%)**	
Asian	1 (2.6)
Black	5 (12.8)
Indigenous / Native American	1 (2.6)
White	31 (79.5)
No answer	1 (2.6)
**Ethnicity (%)**	
Hispanic	1 (2.6)
Non-Hispanic	38 (97.4)
**Clinical Characteristics**	
Median Karnofsky Performance Status (KPS) (range)	90 (50–100)
Prior Surgery (%)	14 (35.9)
Histology confirmed (%)	14 (36.8)
Median Follow-Up (months) (range)	122.2 (12.2–222.1)

**Table 2:** Meningioma symptoms at presentation (*n* = 39)

Presenting Symptoms (%)	
Diplopia	21 (53.8)
CN VI palsy	16 (41.0)
CN III palsy	12 (30.8)
Headache	11 (28.2)
Blurry vision	7 (17.9)
Numbness/paresthesia	7 (17.9)
Ptosis	6 (15.4)
Visual field loss	4 (10.3)
Other ^a^	10 (25.6)

^a^ Other symptoms include proptosis (*n* = 2), vertigo (*n* = 1), CNV deficits (*n* = 1), fatigue (*n* = 1), hair loss (*n* = 1), seizure (*n* = 1), decreased taste (*n* = 1), throat fullness (*n* = 1), tearing (*n* = 1)

### Treatment characteristics

All patients were treated with fSRT, with a median total dose of 54.0 Gy (range 50.0–54.0 Gy) delivered in a median of 30 fractions (range 25–30 fractions) (Table 3). Median dose per fraction was 1.8 Gy (range 1.8–2.0 Gy). Median target volume was 10.0 cc (range 3.6–18.3 cc).

The median of the mean PTV dose was 55.9 Gy (range 51.3–63.8 Gy). Median maximum OAR doses were within typical constraints.

**Table 3:** Treatment characteristics (*n* = 39)

Treatment Characteristics	
Median dose (Gy) (range)	54.0 (50.0–54)
Median number of fractions (range)	30 (25–33)
Median dose per fraction (Gy) (range)	1.8 (1.8-2.0)
Median target volume (cc) (range)	10.0 (3.6–18.3)
Median PTV Dmean (Gy) (range)	55.9 (51.3–63.8)
Median PTV Dmax (Gy) (range)	57.1 (52.2–66.7)
Median PTV Dmin (Gy) (range)	53.4 (48.1–56.4)
**Median OAR Dmax (Gy) (range)**	
Optic Chiasm	52.9 (32.5–56.4)
Brainstem	53.8 (12.2–62.0)
L Optic	51.6 (6.1–57.8)
R Optic	46.1 (4.2–58.8)
L Eye	3.7 (0.6–10.6)
R Eye	3.3 (0.5–54.6)

### Local control

**Fig. 2:**
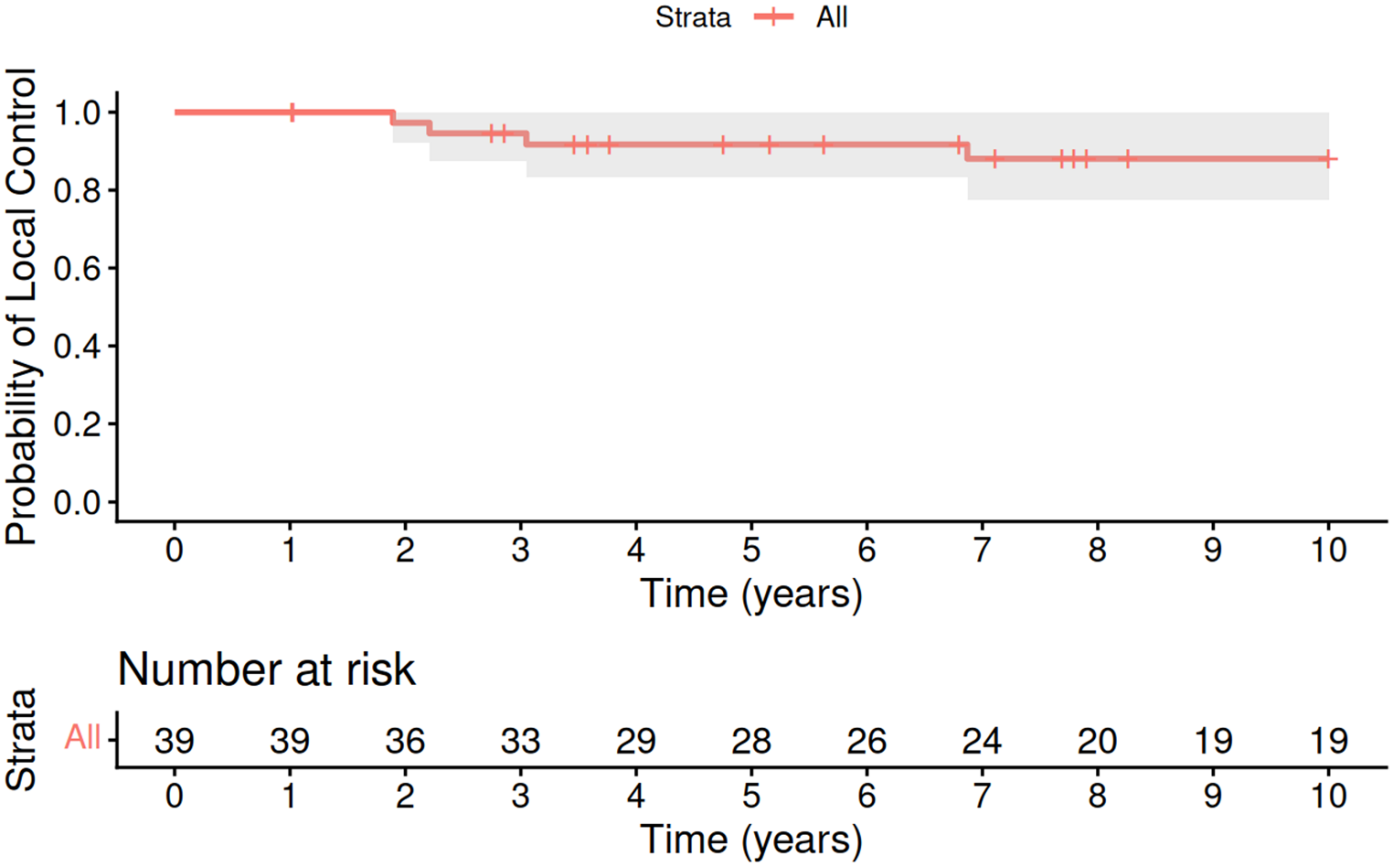
Local control at 1, 3, 5, and 10 years (*n* = 39) with 95% confidence interval

Imaging follow-up was available for 39 patients at 1 year, 35 patients at 3 years, 31 patients at 5 years, and 23 patients at 10 years. Local control rates at 1, 3, 5, and 10 years were 100%, 94.6%, 91.7%, and 88.1%, respectively (Fig. 2). Characteristics and subsequent outcomes for patients with local failure (*n* = 4) are described in Table 4. Salvage modality selection was not standardized.

**Table 4:** Characteristics and subsequent management of patients with local failure (*n* = 4)

Age (Sex)	Prior Surgery (Y/*N*)	Time to Failure (months)	Failure Location	Target Volume (cc)	Salvage Treatment	Subsequent Outcome
79 (M)	Y	27	In-field	13.9	None	Worsening extraocular movement Death at 38 months after RT due to meningioma
62 (F)	Y	82	Marginal	9.6	fSRT 50.4 Gy in 28 fx	Clinically stable after salvage RT
47 (F)	N	22	In-field	5.5	None	Initial symptomatic improvement, then clinically stable
46 (F)	Y	37	Marginal	3.6	fSRT 52.2 Gy in 29 fx	Worsening headaches prior to salvage RT Radiographic improvement without toxicity from salvage RT

### Symptom response

Clinical follow-up was available for 39 patients at 1 year, 33 patients at 3 years, 30 patients at 5 years, and 21 patients at 10 years. Of 39 patients, 26 (66.7%) demonstrated clinical improvement following fSRT, 9 (23.1%) remained clinically unchanged, and 4 (10.3%) experienced worsening without subsequent improvement. All 26 patients who improved had already demonstrated improvement by 36 months, and no patient first achieved improvement at a later follow-up interval.

CN III palsy demonstrated the highest rate of clinical improvement (11/12, 91.7%), followed by blurry vision (6/7, 85.7%), ptosis (5/6, 83.3%), and CN VI palsy (13/16, 81.3%). Diplopia, the most common presenting symptom, improved in 14 of 21 patients (66.7%), remained unchanged in 4 (19.0%), and worsened in 3 (14.3%).

Among the four patients with symptomatic worsening after fSRT, three (7.8%) had worsened symptoms at < 36 months, with two of these patients complaining of double vision and the other experiencing vision loss. Two (6.9%) reported worsened symptoms from 36 to 60 months, one with sustained double vision and another with headaches. One (4.2%) reported worsening diplopia with CNVI palsy from 61 to 120 months. From 121 to 180 months, one (8.3%) reported worsening of blurry vision, CNIII and CNVI nerve palsies, and ptosis.

Among 25 patients without prior surgery, 17 (68%) reported improvement, compared to 9 (64.3%) of the 14 patients with prior surgery. Two (8%) of the 25 patients without prior surgery had worsened symptoms, one from 61 to 120 months after treatment and one from 121 to 180 months after. Five (35.7%) of 14 patients with prior surgery experienced worsened symptoms, which occurred for 3 patients at < 36 months and for 2 patients from 37 to 60 months.

### Toxicities

Twenty-nine patients (74.4%) experienced toxicity attributed to treatment during clinical follow-up (Table 5). The most reported toxicity was fatigue (61.5%). Of those with any toxicity, 25 had Grade 1 toxicity only, 3 had Grade 2 toxicity, and 1 experienced a Grade 3 seizure. No Grade 4 or 5 toxicities occurred.

**Table 5:** Treatment-related toxicities (*n* = 39)

Toxicities (%)	
Any Toxicity	29 (74.4)
CTCAE Grade 1 Toxicity Only	25 (64.1)
CTCAE Grade 2 Toxicity	3 (7.7)
CTCAE Grade 3 Toxicity	1 (2.6)
Fatigue	24 (61.5)
Headache	6 (15.4)
ICA stenosis	3 (7.7)
Vision changes / blurry vision	2 (5.1)
Diplopia	2 (5.1)
Alopecia	2 (5.1)
Trouble sleeping	2 (5.1)
Ptosis	2 (5.1)
Seizure, Grade 1	1 (2.6)
Seizure, Grade 3	1 (2.6)
Grade 2 radiation necrosis	1 (2.6)
Other ^a^	4 (10.2)

^a^ Other includes numbness (*n* = 1), nausea/vomiting (*n* = 1), dizziness (*n* = 1), proximal muscle weakness (*n* = 1)

## Discussion/conclusions

This single-institution retrospective cohort study provides important long-term data on radiographic, clinical, and toxicity outcomes of fSRT (fractionated stereotactic radiotherapy) for symptomatic cavernous sinus meningiomas. The findings build upon prior evidence supporting fSRT as an acceptable standard-of-care approach for patients who are poor candidates for surgical resection or SRS due to tumor size or proximity to neurovascular structures. Over one-third of patients in this study had undergone prior surgical resection, providing additional clinical evidence that fSRT offers effective and durable local control when used in either the adjuvant or salvage setting. Our cohort demonstrated high rates of local control at 1, 3, 5, and 10 years of radiographic follow-up. These outcomes are consistent with prior reports citing high rates of long-term radiographic control following fSRT for cavernous sinus meningiomas [15]. Although the subset of patients with follow-up extending to 15 years or more was smaller than the original cohort, no local failures were observed in this group. Limited data are available in the literature on such long-term follow-up, so this still contributes crucial information to build upon existing studies by Correa et al. and Milker-Zabel et al., which followed fSRT patients up to 10 years [19, 20].

Clinical symptom response mirrored the favorable radiographic outcomes, with most patients reporting symptomatic improvement or stability through long-term follow-up. No existing studies have continuously evaluated symptom control throughout the duration of follow-up. Literature reports of clinical follow-up outcomes vary significantly in methodology and outcome measures, but low rates of clinical worsening have been consistently noted following fSRT, which aligns with this study’s findings [15]. Although the sample size is small, a greater proportion of patients with prior surgery had symptom worsening compared to those without prior surgery. Among these patients, those with prior surgery experienced worsening within 5 years of treatment and patients without prior surgery only experienced worsening more than 5 years after treatment.

The toxicity profile in our cohort was also favorable, with most treatment-related toxicities limited to Grade 1 or 2. Fatigue was the most common reported toxicity overall. One patient developed Grade 3 seizures, and one developed confirmed G2 radionecrosis, yielding a low rate of serious adverse events. These findings support previous literature indicating that fSRT provides a lower risk of complications compared to surgical resection or SRS for large meningiomas or those meningiomas abutting neurovascular structures [12, 14, 15].

Several prior studies have examined the use of fSRT for cavernous sinus meningiomas, though there are important differences that highlight the contribution of the present study. For example, similar studies by Brell et al. and Litre et al. evaluating fSRT for cavernous sinus meningiomas are limited by median follow-up of less than 5 years [17, 18]. Another cohort study by Metellus et al. included cavernous sinus meningiomas of WHO Grades 1 through 5, ultimately including less than 20 patients with Grade 1 meningiomas, the focus of the present study [19]. Correa et al. and Milker-Zabel et al. have published similar studies that follow benign cavernous sinus meningioma cohorts of 89 and 57 patients, respectively, for up to 10 years after RT [20, 21]. However, the median follow-up for both of these studies was less than 7 years, and it is unclear exactly how many patients were followed through 10 years. The limitations of these comparable studies illustrate the relative lack of long-term data for cavernous sinus meningiomas treated with fSRT. This includes the absence of information about long-term symptom response, as no existing studies continuously report symptom response throughout the entire duration of follow-up.

This study is limited by its retrospective design and modest sample size with loss to follow-up. Requiring a minimum of 1 year follow up may exclude patients who disengaged from care due to clinical worsening, potentially overestimating local control and symptom improvement. Less than half of the patients had histological confirmation, introducing risk that other benign tumors were misclassified as cavernous sinus meningioma. Also, cavernous sinus invasion grading (e.g., Sekhar classification) was not systematically recorded, precluding subgroup analysis by tumor extent and limiting comparability with series that report this variable. Radiographic evaluation would have been strengthened by the use of Response Assessment in Neuro-Oncology (RANO) meningioma or volumetric criteria, which could not be retroactively analyzed for all patients.

Furthermore, symptom duration prior to treatment was not captured in our database; its absence prevents analysis of whether earlier intervention is associated with improved neurological recovery. Determining symptom improvement through clinician documentation rather than standardized neuro-ophthalmological examinations introduces potential reporting variability. Additionally, more specific information on timing and duration of reported toxicities would improve this study’s evaluation of treatment safety, particularly with respect to acute, subacute, and late effects of radiation in this population. Despite these limitations, the long follow-up period, and reporting of radiographic, symptomatic, and toxicity outcomes enhance this study’s contributions to the literature, which is currently limited by a relative lack of long-term data for fSRT in this population.

In summary, this study reports favorable long-term radiographic, clinical, and safety outcomes of fSRT for symptomatic cavernous sinus meningiomas. Given its advantages when treating larger tumors or those abutting sensitive neurovascular structures, fSRT remains a useful treatment modality for those patients. Prospective studies with structured outcome assessments are warranted to further establish safety and efficacy.

## Data Availability

The datasets generated during and/or analyzed during the current study are available from the corresponding author on reasonable request.
